# Understanding the Level of Integration in Existing Chemical Clusters: Case Study in the Port of Rotterdam

**DOI:** 10.1007/s43615-024-00410-5

**Published:** 2024-10-09

**Authors:** Michael Tan, Paola Ibarra-González, Igor Nikolic, Andrea Ramírez Ramírez

**Affiliations:** 1 Department of Engineering Systems and Services, Faculty of Technology, Policy and Management, Delft University of Technology, Jaffalaan 5, Delft, 2628 BX The Netherlands; 2 Department of Multi-Actor Systems, Faculty of Technology, Policy and Management, Delft University of Technology, Jaffalaan 5, Delft, 2628 BX The Netherlands; 3 Department of Chemical Engineering, Faculty of Applied Sciences, Delft University of Technology, Van der Maasweg 9, Delft, 2629 HZ The Netherlands

**Keywords:** Petrochemical clusters, Complex networks, Network analysis, Performance evaluation, Industrial clusters

## Abstract

The petrochemical industry is composed of several interconnected processes that use fossil-based feedstock for producing chemicals. These processes are typically geographically clustered and often belong to different parties. Reducing the environmental impacts of the petrochemical industry is not straightforward due to, on the one hand, their reliance on fossil fuels for energy and as a feedstock and, on the other hand, the significant level of interconnected energy and material flows among processes. Current methods for analyzing changes to existing processes cannot capture the multitude and level of interactions. The goal of this paper is to create a model of a petrochemical cluster and analyze its physical characteristics and performance. This paper addresses this goal by developing an assessment method that combines process simulations, multiplex graph analysis, and key performance indicators. The method is applied to a case study based on the petrochemical cluster in the Port of Rotterdam, resulting in a uniquely highly detailed model of a petrochemical cluster. The network analysis results show that only some of the processes are very interconnected. From the performance analysis, it can be observed that the olefins process is the most carbon-intense and has high CO_2_ emissions. Additionally, the results showed the importance of considering existing interconnections when assessing the current performance of existing petrochemical clusters or the performance due to future changes to chemical processes. For instance, some changes would occur to an industrial cluster by introducing alternative carbon sources, such as biomass or CO_2_.

**Supplementary Information:**

The online version contains supplementary material available at 10.1007/s43615-024-00410-5.

## Introduction

Petrochemical processes are highly dependent on fossil-based inputs both as a material feedstock and energy source and, thus, are globally one of the top three CO_2_-emitting sectors [1]. Typically, to improve their individual performance, these processes operate in close geographic proximity in so-called petrochemical clusters, where they share heat, water, electricity, and multiple materials. Therefore, modifying or replacing existing processes with new process technologies can cause cascading effects beyond the location where the changes are made. For example, removing a process currently sharing steam with other processes increases the amount of steam imported from fossil sources, thus resulting in an overall increase of CO_2_ emissions at the cluster level. These types of interactions are frequently assumed to be negligible in aggregated models, often used to identify potential pathways for the transition of the chemical industry. For instance, Meng et al. [2] identified planet-compatible pathways for transitioning the chemical industry. However, they did not include the potential impacts of plant-level changes in their analysis due to a lack of openly available data. Assessing the magnitude of these impacts requires first understanding the complexity and structure of the various interactions among utility and chemical plants within existing petrochemical clusters.

Petrochemical clusters can be regarded as complex adaptive systems, and as such, methods like complex network analysis have been used to understand and analyze them [3]. For example, Domenech and Davies [4] used complex network analysis to study the exchange of materials, water, energy, and knowledge in the Kalundborg industrial cluster by determining the density, degree centrality, betweenness centrality, and closeness centrality of the unweighted exchange network. The results identify the role and importance of each company within the industrial cluster. Similarly, Zhang et al. [5] considered material, energy, and information exchange networks in ten different eco-industrial parks in China and around the world. Their approach, based on unweighted interconnections between industrial plants, allowed them to determine differences in structural characteristics, such as density and degree, among the different industrial parks. Han et al. [6] also used an unweighted physical connections approach to understand the relations and dynamics of flows between companies in the Xinfa Industrial Park over a period of ten years. The authors used measures of centrality and density to identify the most important companies and the changes in interdependencies due to the addition of companies. Song et al. [7] studied the occurrence of symbiotic relationships in the Gujiao Eco-Industrial Park and used the density of complex networks to show that the symbiotic network in the industrial park is in early development and requires further optimization to increase exchanges between companies. More recently, Liu et al. [8] found a similar result for Nanjing Jiangbei New Materials High-Tech Park by assessing density and centrality indicators. Xie et al. [9] used network analysis to demonstrate the increase of symbiotic relationships in the Qinghai Salt Lake Industrial Park from 2014 to 2018.

There are, however, two main limitations to those studies. The first is the assumption that the connections between companies are equally important, i.e., using an unweighted, single-layer network approach. In reality, however, connections differ in quantity and quality (e.g., the amount and type of shared flows). The potential impact the removal or modification of a process and associated flows can have in the overall cluster can cause different impacts of different magnitudes. For example, reducing the quantity of steam exchanged between two companies may have a different impact than changing the steam’s quality (temperature, pressure). Additionally, it is important to distinguish between the different types of interactions, as companies can share more than one flow (e.g., material, energy). Most studies aggregate all the different types of interactions into a single layer. Multi-layered graph analysis allows replicating a network’s nodes over different layers, with each layer representing a particular aspect of a connection between the nodes. Domenech and Davies [4] used this approach in their Kalundborg paper, allowing them to identify the importance of the nodes for each layer of the exchange network and how their disruption could potentially affect the network. As the modification or removal of connections can have different impacts based on their type of interaction, a multi-layered approach should be used when analyzing petrochemical systems.

The second limitation is that complex network analysis alone cannot provide meaningful metrics to determine an industrial cluster’s environmental, economic, and technical performance. Therefore, additional metrics are required to assess a cluster’s current performance, identify areas of improvement, and propose potential modifications. For instance, Jacobsen [10] assessed the economic benefits and reduction in emissions due to industrial symbiosis (IS) in the Kalundborg eco-industrial park by comparing it to a theoretical situation with no symbiotic exchanges. A similar approach has been used to study the reduction of the global warming potential (GWP) for the Xinfa Group industrial cluster [11], the GWP and energy usage for the Kymi eco-industrial park [12], the resource efficiency and environmental performance of the chlor-alkali industry of Shanghai Chemical Industry Park [13], as well as the economic and environmental benefits in the Midong Chemical Industrial Park due to IS [14]. These studies showed reduced GWP, economic savings, and lower energy usage as some of the benefits associated with IS. Lyu et al. [9] developed two algorithms to maximize the symbiotic network in industrial clusters. They found that significant environmental benefits could be achieved by increasing industrial symbiosis.

However, Martin et al. [15] found that IS will not always perform better for all environmental indicators, as illustrated when examining the environmental performance of integrated bioethanol and biogas production at the Händelö Eco Industrial Park. Furthermore, changes in a process may shift the burden from inside to outside the industrial park, for instance, by stopping the production of carbon-intensive materials within the cluster but increasing their import. It is, therefore, important to consider not only direct economic and environmental performances but also indirect ones.

In addition to the two main limitations of the current studies, there is a limitation on data availability. Data on an industrial cluster is usually obtained from interviews with stakeholders and site visits, and the analysis is then performed on this data [16]. However, more detailed process data is required to understand the functioning of each process in an industrial cluster. This detailed process data is only sometimes available as companies are unwilling to share it due to confidentiality issues [17]. Alternatively, this detailed process data can be obtained by creating bottom-up models of the chemical processes using process simulation software.

While complex network analysis and key performance indicators have been used to assess the physical characteristics and understand the environmental performance of a petrochemical cluster, they are insufficient when used as a standalone. Current methods for analyzing the impacts of changes to processes are thus insufficiently able to capture the complex interactions in existing clusters. This study aims to create a detailed model of a petrochemical cluster, analyze the physical characteristics of its exchange network, and assess its performance. Therefore, this study proposes a novel method to assess the performance of existing petrochemical clusters. The method proposed to achieve this objective consists of three main steps: First, a model was developed of a representative petrochemical cluster (RPC), which is used as a case study. Second, network analysis was used to assess the material and energy exchange networks. Third, key performance indicators were identified and used to assess the environmental, economic, and technical performance of the cluster. This article is structure as followed. Section “Methods” presents a detailed description of the method used. Section “Selection and Modeling of Individual Petrochemical Processes Within the Representative Cluster” describes the model of the RPC, which was based on the petrochemical cluster in the Port of Rotterdam (PoR). Section “Results” shows the results of the network analysis and performance evaluation of the RPC, and finally, the conclusions and future outlook are given in Sect. “Conclusions”.

## Methods

**Fig. 1:**
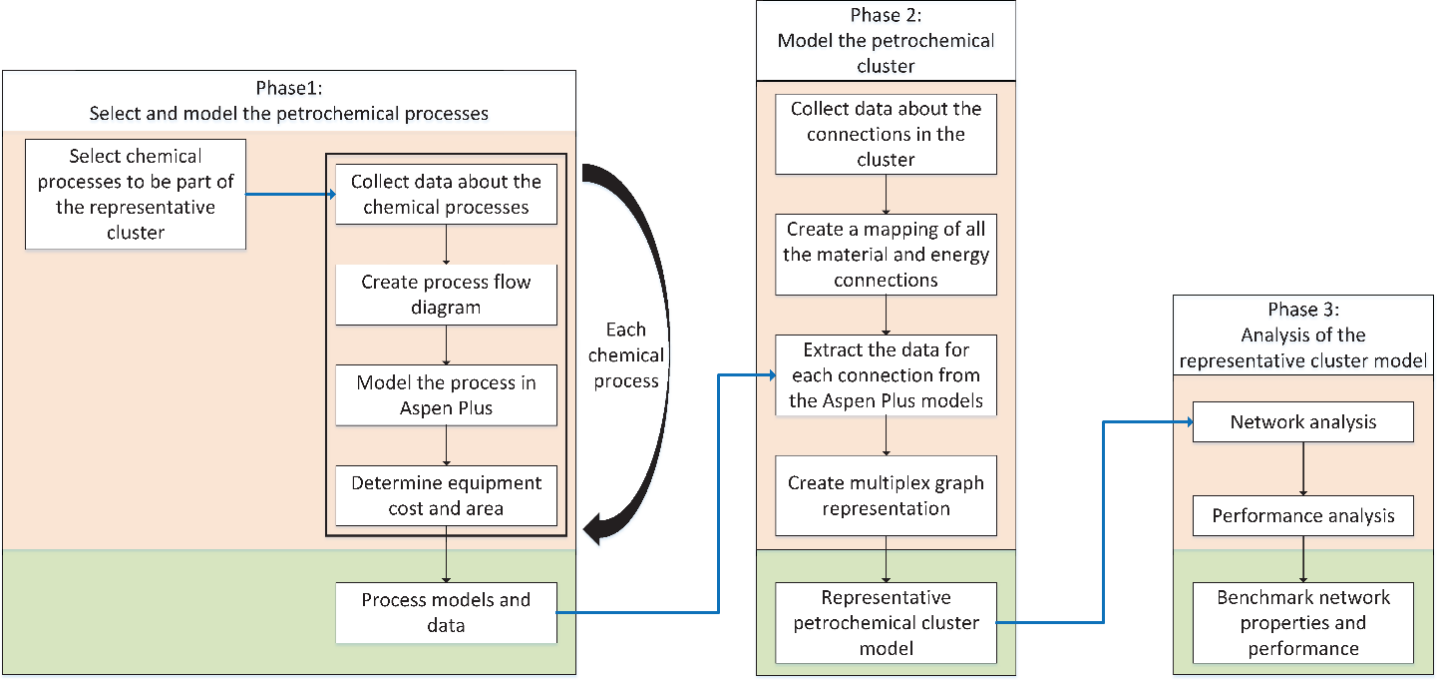
Systematic framework for the development, network analysis and performance evaluation of a RPC

Figure 1 presents an overview of the methodology used in this study. The framework consists of three phases. Phase 1 of the framework considered the definition of an RPC, which consists of selecting chemical processes and their corresponding utility generation units. After the selection, the development of the RPC model involved the collection of data (process descriptions, equipment types, and process conditions) from literature, the translation of the data into individual process flow diagrams, and the modeling of individual chemical processes using a process simulation software (Aspen Plus). From the individual process models, material and energy requirements, resulting products and waste streams, equipment costs, and land footprint were generated. In Phase 2, additional data was collected from publicly available sources to identify existing physical interconnections among processes and utility generation units. The mapping of the interconnections and the mass and energy balances extracted in Phase 1 were used as the core elements for representing the complex network using Python as a modeling and data analysis tool. The third phase consisted of calculating the network properties and the performance indicators inside the cluster. As a result, the RPC model, the complex network properties, and the evaluation of key performance indicators can be used to understand the complexity and operation of petrochemical clusters.

Details on the selection and modeling of the RPC processes, and the Aspen-Python workflow steps used to model and assess the RPC are described in the next subsections.

### Selection and Modeling of Individual Petrochemical Processes Within the Representative Cluster

To set up the RPC, a selection of petrochemical processes producing chemical building blocks (CBB), intermediate chemicals (IC), and end-of-value-chain chemicals (EVC) was made. As a case study, these processes were selected using the current petrochemical cluster in the Port of Rotterdam (PoR). Therefore, the RPC was built to represent the portfolio of products, sizes, and interdependencies existing in the real petrochemical cluster. This representation also includes utility units to provide auxiliary materials (e.g., hydrogen, carbon monoxide, oxygen), electricity, and steam to the chemical processes in the RPC.

For each selected chemical process and utility generation unit, data was gathered from, e.g., openly available reports from the companies, patents, and environmental permits, as well as chemical engineering literature. The goal was to obtain data as close as possible to the actual situation. From this data, detailed process flow diagrams (PFD) were constructed for each chemical process, detailing all the required process steps, i.e., the required pretreatment, reaction, and separation steps to reach the desired product. These PFDs were used to build detailed individual process models of the selected process and utility generation units in Aspen Plus. Equipment lists and mass and energy balances were obtained for each process, providing information such as material requirements, generated waste, product and by-product flows, and utility consumption. An overview of all the required data obtained for each material stream and energy stream is provided in Tables 1 and 2. This data was required for the second phase to ensure that not only a stream’s flow rate would be matched but also its composition, pressure, and temperature. In addition to the material and energy balances, the bare equipment costs were determined for each process using Aspen Process Economics Analyzer.

**Table 1:** Process material data requirements

Data type	Definition	Unit
Mass flow rate	Mass-based flow rate of a stream	ktonne/year
Mass composition	Weight-based fraction of each component in a stream	
Mole flow rate	Mole-based flow rate of stream	mole/year
Mole composition	Mole-based fraction of each component in a stream	
Carbon content	Mass fraction of carbon atoms in a stream	wt%
Pressure	Pressure of a stream	bar
Temperature	Temperature of stream	C
Phase	The phase of a stream, e.g., vapor, liquid, or solid	-

**Table 2:** Process energy data requirements

Data type	Definition	Unit
Type of utility	The type of utility, e.g., electricity, steam, or cooling water	-
Utility demand	The total demand for a type of utility by a chemical process	TJ/year
Utility supply	The total supply of a type of utility by provided by a chemical process or a utility generation process	TJ/year
Energy flow rate	The flow of energy between processes in the cluster	TJ/year
Pressure	The pressure of the steam utility	bar
Temperature	The temperature of the steam utility	C

### Modelling a Petrochemical Cluster as a Complex Network

Material and energy interconnections within and among the selected processes were mapped for modeling the RPC. Public available sources were consulted to identify existent material and energy exchanges. This information, combined with the material and energy requirements, allowed the creation of a mapping of all the physical connections in the cluster. More specifically, it includes all the material and energy connections between the processes, the connections between the utility generation units and processes, and any connections to the outside “world” (i.e., outside the system boundaries of the cluster under study). These outside connections include chemicals imported to the cluster and emissions emitted into the environment. For the mapping of electricity and steam interconnections, it was assumed that excess flows would first be used within the generating company and only after they would be exchanged with processes belonging to another company, as reported in public sources. For instance, cogeneration units (CHPs) located within a company were assumed to supply the demand for heat and electricity first by the processes within the company. If the amount supplied was insufficient, it was assumed that extra utilities were imported from the electricity grid or CHPs owned by other companies or located outside the cluster boundary.

**Fig. 2:**
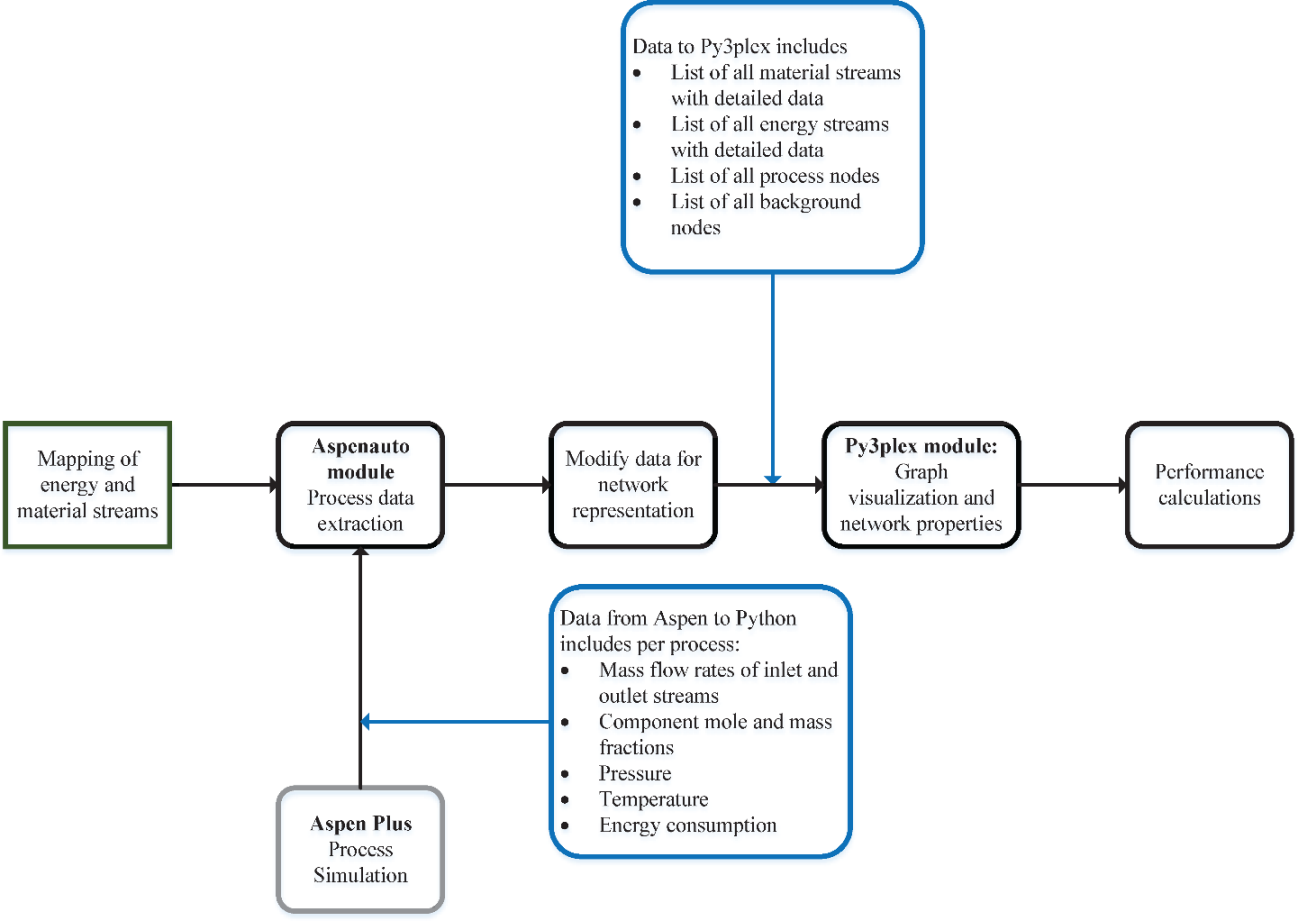
Python workflow used for the modeling and analysis of the representative petrochemical cluster

After modeling the chemical processes in the RPC and identifying interconnections between chemical and utility processes, the physical exchange network of the cluster was modeled. The exchanges of material and energy (physical) flows between processes were represented using multi-layered complex networks, where the processes and utility generation processes are depicted as nodes and the physical connections as links. In Fig. 2, a visual overview of the Python workflow developed for the modeling and analysis of an industrial cluster is presented. Based on the individual models developed in the first phase (see Sect. 2.1) and the mapping of connections, detailed material and energy data was automatically extracted from each Aspen Plus model to Python using an in-house developed Python module. This resulted in a database that includes all the connections within the processes in the RPC, stream data (e.g., mass flow rate, composition, and temperature), and the source and destination of each stream. This data was used to construct a weighted directional multiplex graph representation of the cluster in Py3plex [18]. This specific type of multi-layered graph will be described in more detail in the following subsection.

### Assessing the Petrochemical’s Cluster Complex Network Properties and Performance Indicators

**Fig. 3:**
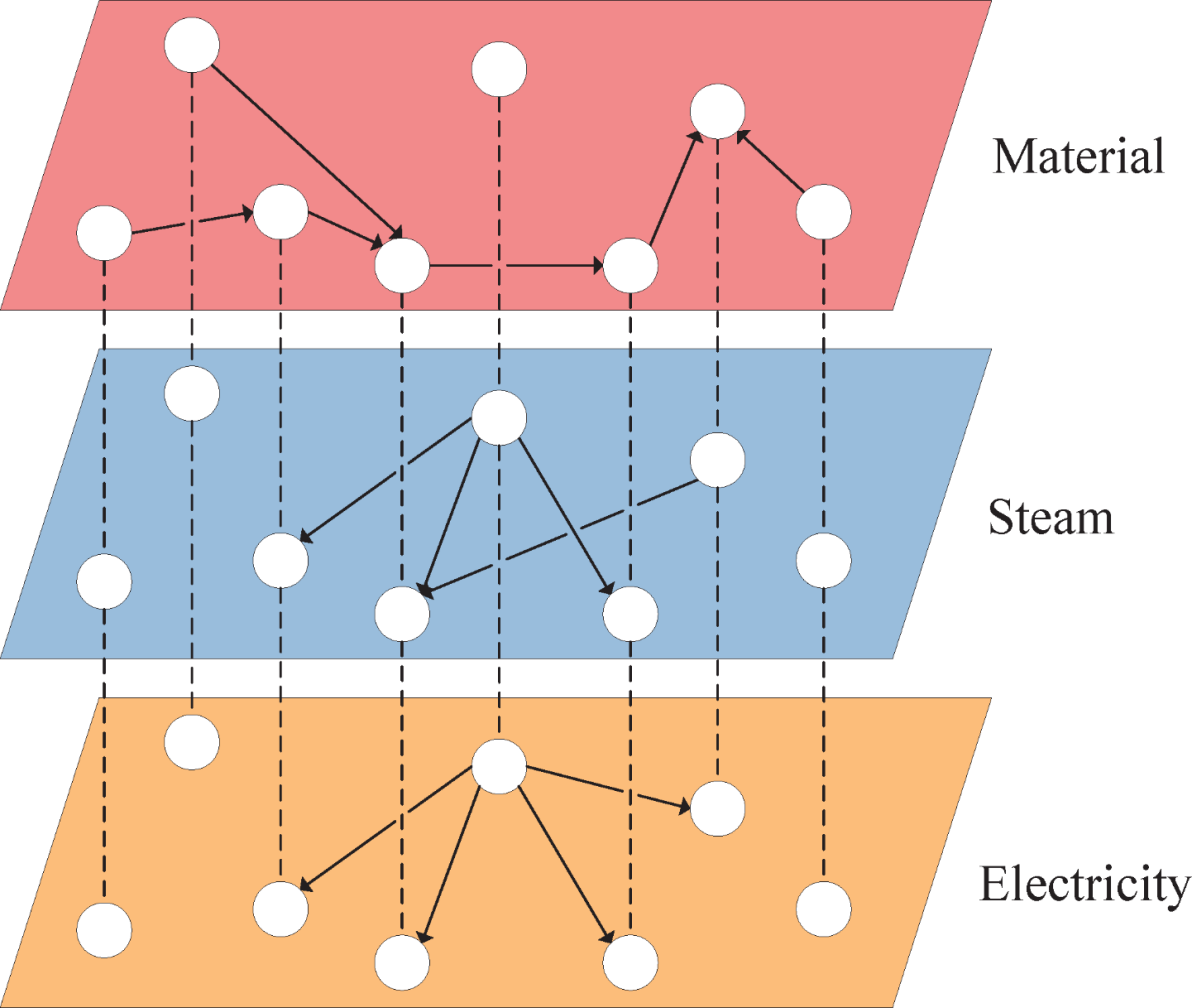
Multiplex graph consisting of a material, steam, and energy layer

The RPC was modeled using a directed weighted multiplex graph representation. As with standard multi-layered graphs, this representation contains separate layers representing different types of interactions between the nodes of the graph. For instance, a material layer containing the exchange of chemicals between processes, a steam layer for the exchanges of thermal energy, and an electricity layer for all electricity exchanges. However, contrary to standard multi-layered graphs where all connections between nodes on separate layers, so-called interlayer connections, are allowed, multiplex graphs only allow interlayer connections to other nodes representing the same process or utility unit [19]. Figure 3 shows a generic example of a multiplex graph consisting of three layers and seven nodes. Each node in Fig. 3 represents a process or utility unit and is present in each layer of the multiplex graph, with the difference between the layers being the connections present on each layer of the multiplex graph. Extending this approach to a complete industrial cluster allows us to determine the importance of processes or utility generation units for each graph layer.

Each interconnection was assigned a weight representing the quantity transferred between nodes. For instance, in the material layer, the weights represent the carbon mass flow rate, while in the steam and electricity layers, the weights represent the energy flow rate. These weights were normalized by dividing them by the highest weight of their respective layers.

The Py3plex module provides visualization options and allows the evaluation of the network properties of a system (e.g., the petrochemical cluster), thereby allowing the physical exchange network to be analyzed. Two complex network properties were selected: degree and degree centrality. The degree describes a node’s importance based on the number of links connected to it. Degree centrality also uses this principle but normalizes the results.

By studying the importance of nodes and links, the potential impacts their removal might have can be understood. The equations used to determine these network properties are provided in the supporting information (SI.1 in Online Resource 1).

### Key Performance Indicators

To understand the functioning and complexity of the cluster, not only its structure but also its economic, environmental, and technical performance need to be assessed. The assessment was performed from gate to gate and, therefore, was based on the Aspen Plus models of the chemical processes and utility generation units. The different processes’ CAPEX are considered as the only economic key performance indicator. It provides a benchmark of the money invested in each process within the RPC and the whole cluster. It is calculated based on the bare equipment costs determined for each process in previous steps. The equations used for calculating the CAPEX are presented in the Supporting Information (SI.2 in Online Resource 1).

The environmental performance was assessed considering only the CO_2_ emissions (direct process CO_2_ emissions and energy-related CO_2_ emissions). It should be noted that this approach can be extended to assess other indicators, such as water consumption and land requirements. Equation (1) calculates the direct CO_2_ emissions of a process or utility generation unit:

1 $$\:C{O}_{2,p}^{Emis}=\sum\limits_{w=1}^{{N}_{w}}{m}_{C{O}_{2},w}^{Waste}$$

where, $\:{m}_{C{O}_{2},w}^{Waste}$ is the mass of CO_2_ being emitted to the environment. Similar to specific energy consumption (SEC) [20], where SEC is defined as the energy consumption per tonne of product, we defined the CO_2_ intensity as the CO_2_ emitted per ktonne of carbon in the product. Based on the direct CO_2_ emissions the CO_2_ intensity of a process or utility generation unit can then be calculated as:

2 $$\:{I}_{C{O}_{2}}=\frac{C{O}_{2,p}^{Emis}}{{\sum\:}_{p=1}^{{N}_{P}}{m}_{Carbon,p}^{{Pr}oduct}}$$

Where $\:{m}_{Carbon,P}^{Product}$ is the mass of carbon in a process’ product stream $\:p$.

The total electricity and steam consumption, steam intensity, and carbon feedstock efficiency were evaluated for the technical performance. The carbon efficiency $\:{\eta\:}_{Carbon}$ is used to evaluate the efficiency of the cluster in transforming the carbon in the feedstock into the desired products. It is defined as:

3 $$\:{\eta\:}_{Carbon}=\frac{\sum\:{m}_{Carbon,p}^{{Pr}oduct}}{\sum\:{m}_{Carbon,f}^{Feed}}$$

Where, $\:{m}_{Carbon,P}^{Product}$ is the mass of carbon in product stream $\:p$, and $\:{m}_{Carbon,f}^{Feed}$ is the mass of carbon in feed stream $\:f$.

The total steam consumption of the cluster $\:{E}_{Steam,type}^{Total}$ per type of steam (very low/low/medium/high pressure steam) was calculated using Eq. (4):

4 $$\:{E}_{Steam,type}^{Total}=\sum\limits_{i=0}^{n}{E}_{Steam,type}^{{Pr}ocess,i}$$

Where, $\:{E}_{Steam,type}^{Process,i}$ is the steam consumption of a steam type in process $\:i$.

The steam intensity $\:{I}_{Steam}^{Process,i}$ of process $\:i$ provides a normalized steam consumption, allowing processes with different production capacities to be compared and was calculated using Eq. (5) The total electricity demand of the cluster $\:{E}_{Electricity}^{Total}$ was calculated by using Eq. (6).

5 $$\:{I}_{Steam}^{{Pr}ocess,i}=\frac{{\sum\:}_{type=LLPS}^{HHPS}{E}_{Steam,type}^{{Pr}ocess,i}}{{m}_{Carbon}^{{Pr}ocess,i}}{I}_{Steam}^{{Pr}ocess,i}$$

6 $$\:{E}_{Electricity}^{Total}=\sum\limits_{i=0}^{n}{E}_{Electricity}^{{Pr}ocess,i}$$

Where $\:{m}_{Carbon}^{Process,i}$ is the total mass of carbon in the products in process $\:i$ and $\:{E}_{Electricity}^{Process,i}$ is the electricity demand of process $\:i$. Each process’s steam, cooling, and electricity consumption were retrieved from the Aspen Plus process models.

## Case Study

As described in the methodology, an in-house developed model of an RPC was built based on the cluster located in the Port of Rotterdam (PoR). This model mimics the type of product portfolios, production capacities, interactions, and interconnections within the PoR industrial cluster. For the model development, several chemical processes producing a variety of chemical building blocks (CBBs), intermediate chemicals (ICs), and end-of-value-chain chemicals (EVCs) were selected. The definition of the case study was based on publicly available information, such as reports [21]. The processes were selected to include several chemicals along different value chains existing in the PoR’s product portfolio and to mimic the range of production capacities.

The refineries were considered outside of the system boundaries, and only the relevant refinery’s product streams and processes involved in the conversion of CBBs (e.g., ethylene and benzene) into EVCs (e.g., ethylene glycol and polyvinylchloride) were selected. The selection resulted in a cluster of 30 chemical processes producing 52 chemicals. To operate the processes, utilities such as steam, electricity, and hydrogen are required; thus, 20 utility generation units were included. These 30 chemical and 20 utility generation processes were assigned to companies (from A-S) to match the real-life counterparts as closely as possible.

**Fig. 4:**
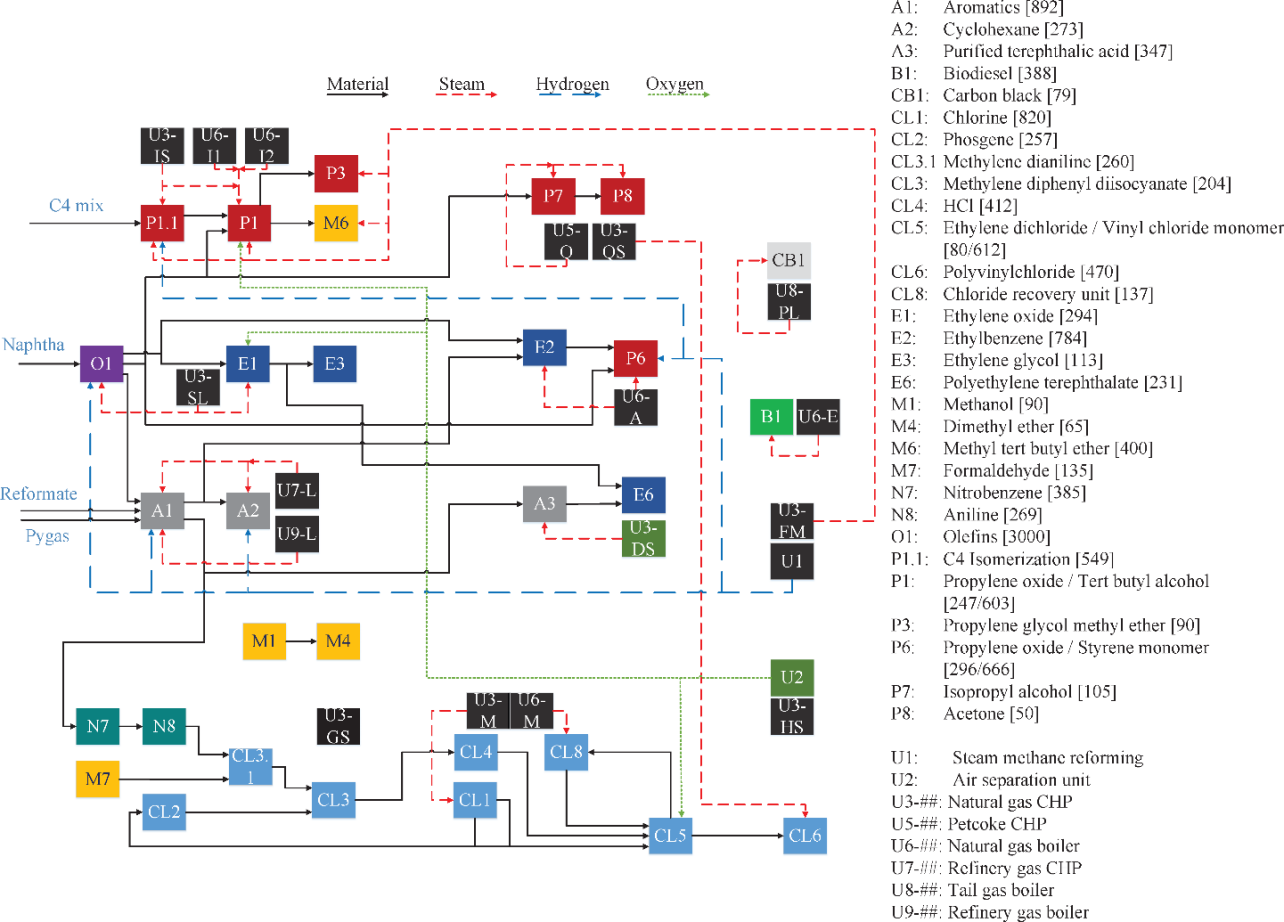
Overview of the representative petrochemical cluster, its main interconnections, and production capacities

Figure 4 shows an overview of the cluster’s processes, utility generation units, and their connections. Note that the purpose of this figure is only to illustrate the value chains present in the cluster and their most important connections. In Fig. 4, the production capacity of the chemical processes is shown in brackets in ktonne/y. This production capacity is based on the mass flow rate of naphtha for the olefins, while it is based on the mass flow rate of the main products for all the other processes. A complete list of all the chemical processes, their products, production capacities, and company classification is presented in Table A.1 of Online Resource 1. The list of utility generation processes of the representative cluster is found in Table A.2 of Online Resource 1.

The selected processes were grouped into sub-clusters, where all the processes are related to or part of a value chain. An example of a sub-cluster is the ethylene sub-cluster, depicted in dark blue in Fig. 4, composed of E1 ethylene glycol, E2 ethylbenzene production, and E6 Polyethylene terephthalate processes. It contains all processes based on the CBB ethylene or its derivatives. Furthermore, several utility generation units (tagged as U1, U2, and U3-U9##, with the ## used to identify each unique utility process), such as CHPs and boilers, were added. These utility units were also modeled using openly available sources to reflect existing units in the PoR as closely as possible.

Based on the methodology presented in Sect. 2, 50 process models were developed as part of project Unraveling (see acknowledgments). Validation of these models was done at two levels: (i) Each model was validated by comparing the material requirements and the energy consumption with public sources; (ii) The total CO_2_ emissions at the company level were compared to environmental permits and the CO_2_ emission register [22]. As indicated in the methodology, only CO_2_ emissions into the atmosphere were considered. Furthermore, the treatment of waste streams such as wastewater and solid waste was outside the cluster boundaries and was not considered in the cluster performance analysis. Using the methodology explained in Sect. 2, a multiplex graph representation of the RPC was created. The following section will present the results of these network properties and performance indicators.

## Results

### Complex Network Properties of the Representative Cluster

**Fig. 5:**
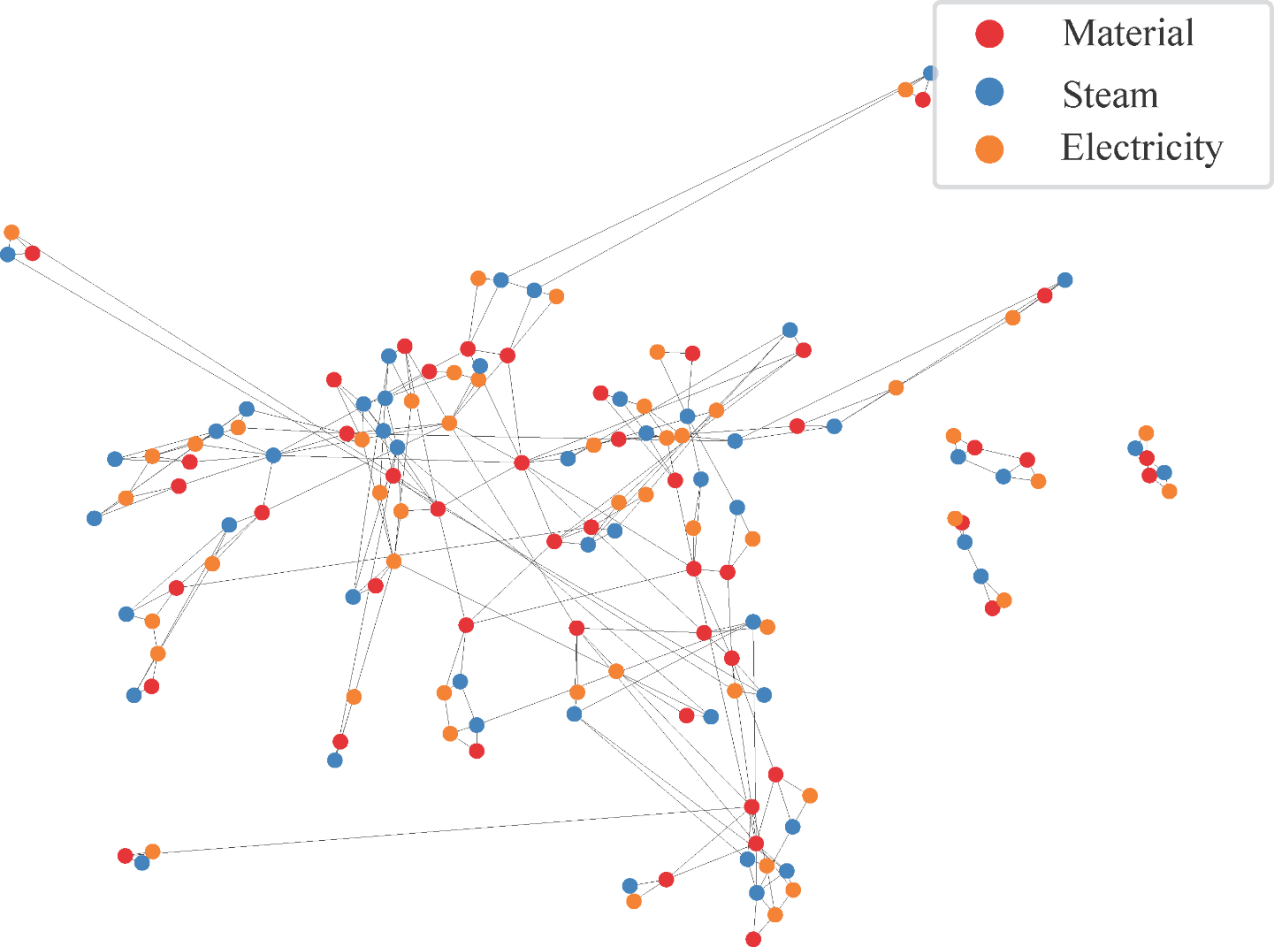
Hairball representation of the representative petrochemical cluster

The multiplex graph of the RPC consists of 50 nodes and 138 links. The links are classified in three layers depending on the different types of physical interactions (material, steam and electricity) occurring in the petrochemical cluster. This leads to the material, steam and electricity layers containing 62, 54 and 22 links, respectively. Figure 5 presents the entire network as a single layer, using a force-directed layout algorithm [23]. Each process or utility generation is represented by three nodes, with the nodes of each layer being represented by a different color. The figure provides an intuitive visual representation of the deep interconnectedness of the processes in RPC, and we can observe that the cluster consists of several centers where the nodes are very interconnected. In addition, there are several groups of nodes that are weakly connected to these centers by either one or two links. This type of structure is commonly found in petrochemical and other types of industrial clusters, where a few processes make up the backbone of the cluster, and the other processes are connected to this backbone [4, 5]. Furthermore, on the right side of Fig. 5, three subsets of nodes are not connected to the main graph, indicating that these processes are operating completely independent of the rest of the cluster. For instance, the methanol and dimethyl ether processes are not connected to the rest of the industrial cluster.

#### Degree Centrality as a Measure of Interdependence

**Fig. 6:**
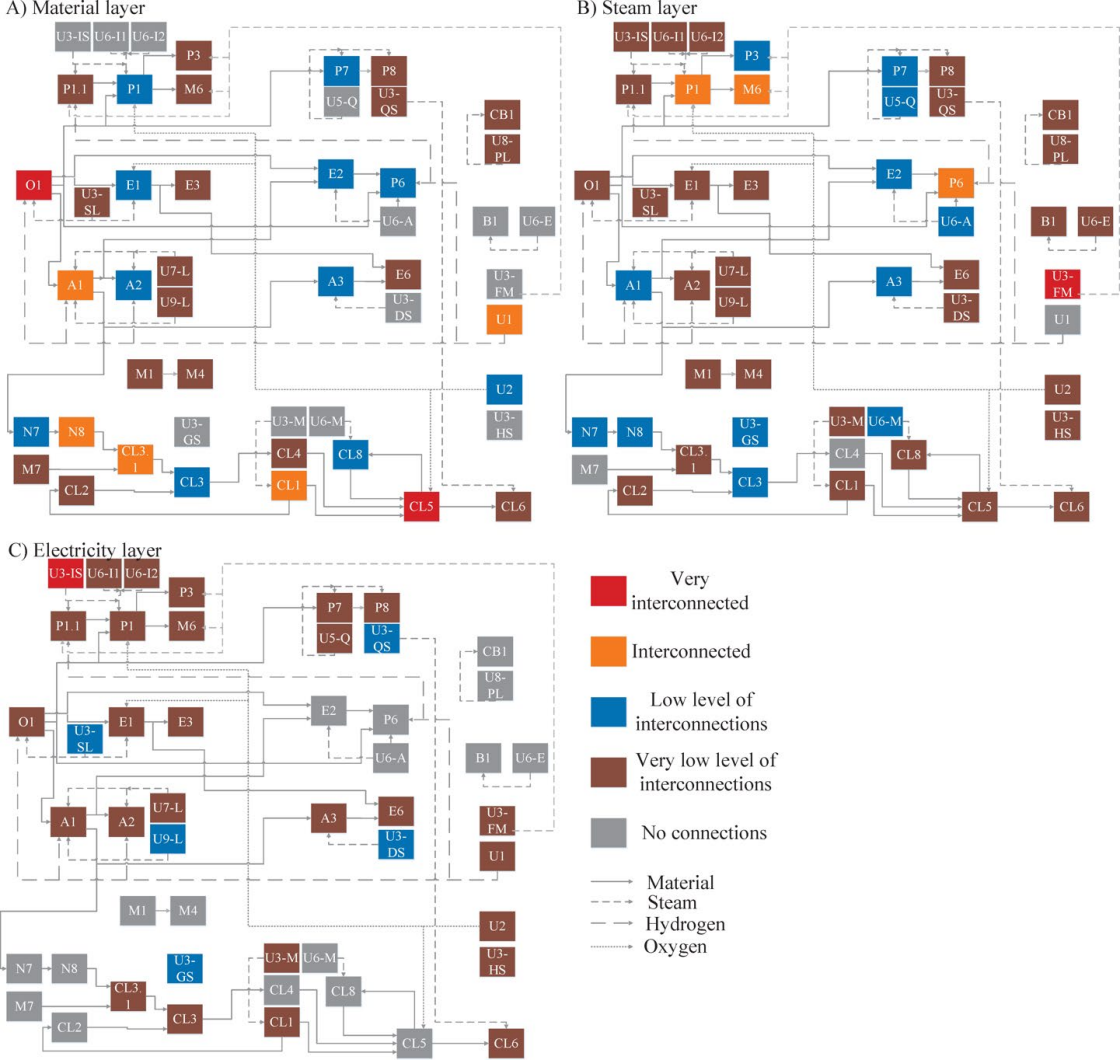
Level of interdependence of the nodes based on their degree centrality

Figure 6 depicts the nodes’ interdependence level based on their degree centrality. It presents the three layers of the multiplex graph of the RPC and demonstrates the interdependence of the material, steam, and electricity flows. Furthermore, most connected nodes(s) are different in each layer, meaning that changes in processes and flows can affect those networks differently. Each layer has several highly interconnected processes, followed by a long tail of weakly interconnected processes. Finally, a large portion of the nodes is only active on some graph layers. For example, several CHPs do not have links on the material layer as they only have inlet and outlet connections to systems outside the cluster boundaries (e.g., with the natural gas provider) and are thus not considered during the network analysis. Therefore, these nodes are only impacted by electricity and steam connection changes.

For the material layer, the olefins plant (O1) and the ethylene dichloride/vinyl chloride monomer process (EDC/VCM, CL5) are the most interconnected processes in the RPC. This is not unexpected as the olefins plant is at the start of most of the value chains in the RPC, and, therefore, it has a large number of outgoing links to other processes. The EDC/VCM process is part of the chlorine sub-cluster (see Fig. 6). It has a large number of incoming material links from other processes that are also part of the chlorine sub-cluster, for example, Chlorine production (CL1) and the methylene diphenyl diisocyanate (MDI, marked by CL3) process. Furthermore, the process has an outgoing material connection to the polyvinylchloride (PVC, marked by CL6) process and a material link containing chlorinated tars to the chlorine recovery unit (CL8). In addition, it can observed that every chemical process has at least one connection to another process. This observation is based on the fact that every chemical process node has the lowest or higher interconnection classification.

When considering the steam and electricity layers, the natural gas CHPs U3-FM and U3-IS are the most interconnected nodes. These nodes provide the required steam and electricity to several processes in their proximity, such as the PO/TBA (P1) and MTBE (M6) processes. Compared to the other two layers, the electricity layer shows fewer interconnections as the CHPs are primarily used to provide electricity to processes within the same company site.

#### Most Important Links

**Fig. 7:**
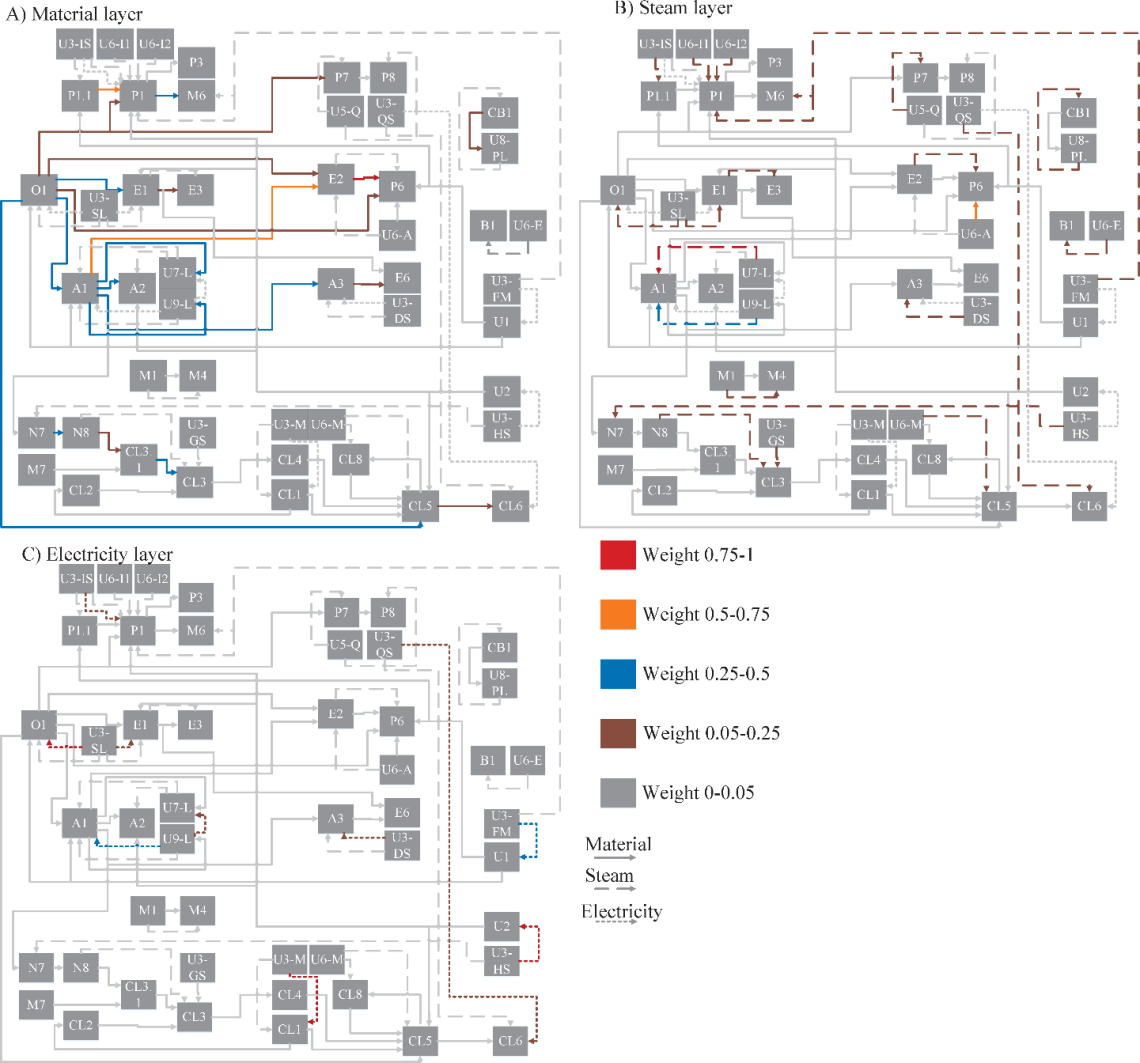
The importance of the links for each layer based on their normalized weights

Figure 7 illustrates the importance of each link for each layer of the graph. It was based on the weights of each link, as highlighted with red, orange, blue, brown, and gray lines. As explained in the methodology, the normalized weights were determined based on the carbon mass flow rate for the material layer and the energy content for the steam and electricity layers. As a result, the links with the highest values are the most interdependent.

There are a few relatively important links on each layer based on the weight of each link. The processes connected by such links are strongly interdependent. Therefore, modifying those processes will significantly impact other processes. Nearly all the important links for the material layer are connected to the olefins plant or the aromatics process. This results from the olefins and aromatics plants being at the start of every value chain and having large outgoing carbon-containing streams to other processes. The link between the EB (E2) and PO/SM (P6) processes is the most important link of the material layer, as both processes have relatively high production capacity, and the PO/SM process uses all EB produced in the cluster. When looking at the steam layer, a large fraction of the links have a relatively low weight, with a small fraction having a high weight. This distribution indicates that most processes are not strongly interdependent through steam connections. For the steam layer, the links that provide steam to the aromatics process (A1) from the refinery gas boiler and to the PO/SM process from a natural gas boiler are the most important. When considering the electricity layer, there are a relatively high number of links with high weights, indicating a strong electricity-based interdependence between several processes and CHPs. The most important links are those supplying electricity to the Chlorine electrolysis process (CL1), the ASU (U2), and the olefins process.

### Performance of the Representative Cluster

#### CAPEX Analysis

**Fig. 8:**
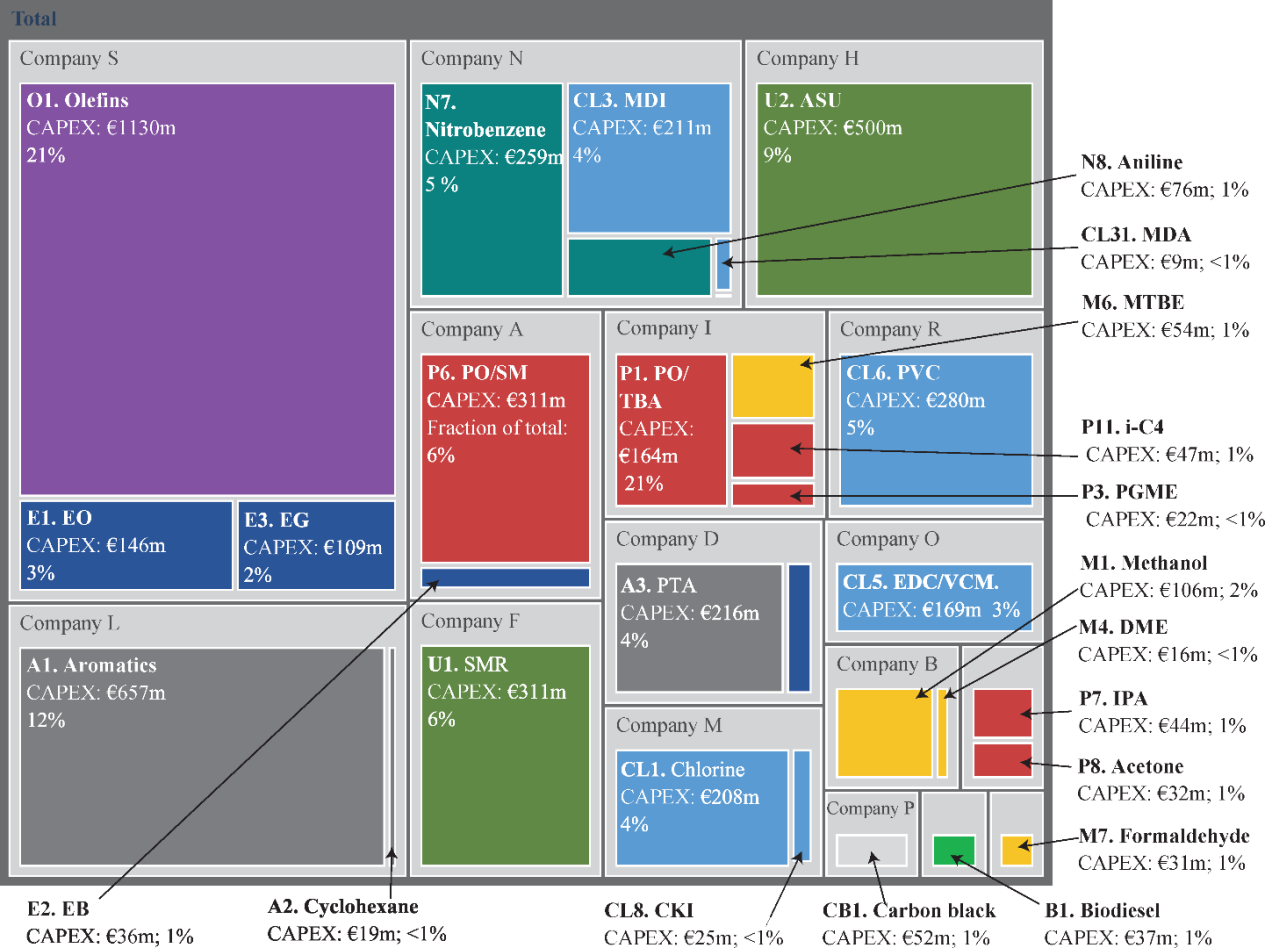
Distribution of the CAPEX in million euros invested in the industrial cluster per process and company

The chemical processes in the RPC required a significant investment by the companies involved in the cluster. The energy transition may require the replacement of those assets. Therefore, analyzing the distribution of the CAPEX investments in the petrochemical cluster is interesting. The total CAPEX was estimated at 5.5 billion euros. Figure 8 shows the distribution of the CAPEX per process and company of the RPC, with the size of each box scaling respectively to its CAPEX. Figure 8 shows that the larger production capacities (a list of production capacities is provided in Table A.1 in the Appendix) and consequentially higher CAPEX are found at the start of the value chains, with the aromatics and olefins plants making up 21% and 12% of the total invested CAPEX in the representative cluster. In contrast, the processes producing intermediate and end-of-value chain chemicals required a much smaller capital investment as these processes are smaller and are focused on producing one or two chemicals. For instance, there are several processes where the invested CAPEX makes up less than 1% of the total CAPEX.

It also shows the large number of investments already done in the cluster and the need to find possible pathways to use those assets when transforming the industrial cluster.

#### Carbon Flow Analysis and Carbon Efficiency

**Fig. 9:**
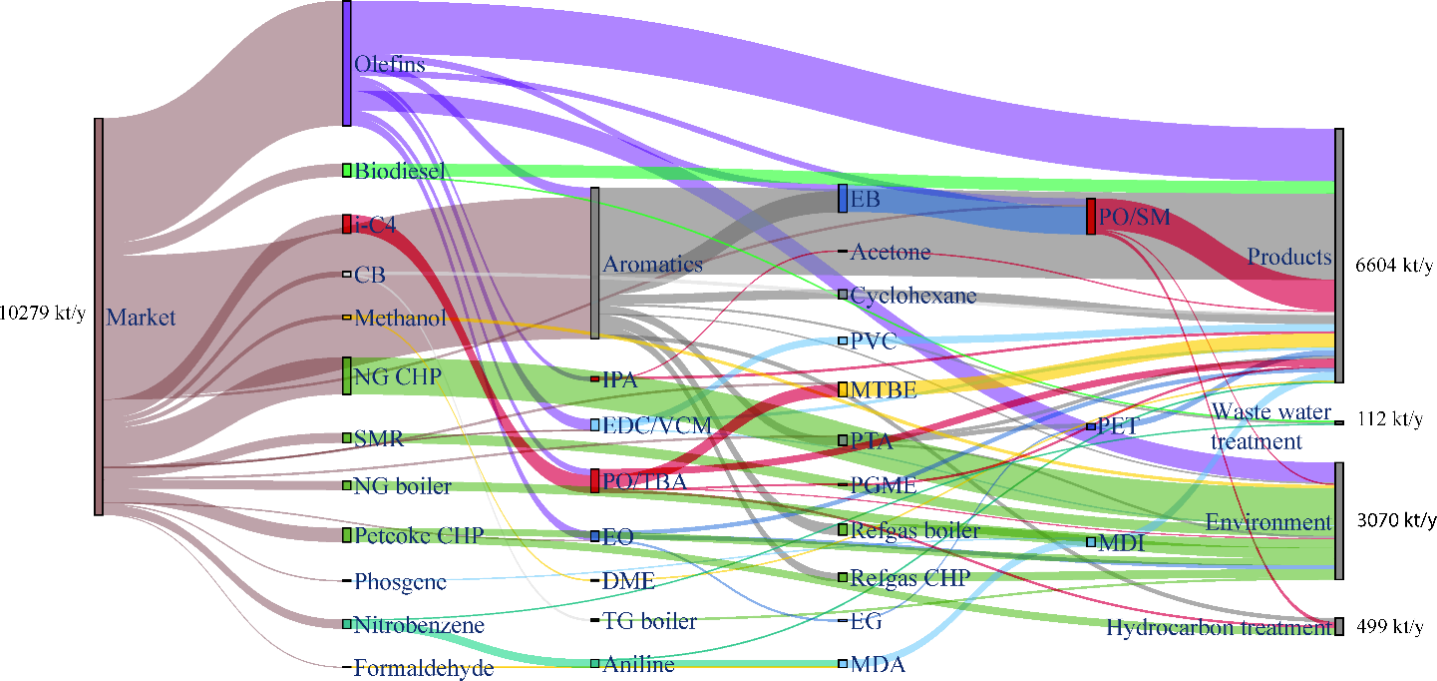
Sankey diagram of the carbon flows in the petrochemical cluster. Carbon flows lower than 15 kt/y are not shown in the figure

The RPC is based on the conversion of carbon-based feedstocks, mostly of fossil origin. Figure 9 shows a Sankey diagram of the carbon flows in the cluster. Figure 9 shows all mass flow connections between processes in the cluster and the outside world. The material flows imported into the cluster are represented by the “Market.” Any carbon emissions in the form of CO_2_ are represented by the “Environment.” The chemical products that are not used within the industrial cluster and instead are exported are defined as “Products.” In comparison, the complex network properties only show interactions between nodes inside the cluster and do not include connections to the outside world.

The carbon efficiency of the RPC is 75%. It should be noted that this is based on the carbon used as a material feedstock and not as an energy source. Most losses of carbon that enters the clusters as raw material feedstock (e.g., natural gas, naphtha) are lost as an atmospheric emission (18%) and to waste treatment (7%). The olefins plant (O1) uses part of its by-products to generate the heat required for the process, while waste streams of the aromatics plant (A1) are used as a fuel source by the refinery gas CHP (U9-L). The aromatics and olefins are the most carbon-intensive processes of the cluster, as they utilize most of the carbon imported, in the form of naphtha and reformate, in the cluster and transform it into CBBs. These CBBs, such as ethylene and benzene, are used across the different value chains of the industrial cluster. However, as expected, only some of the produced CBBs are used inside the cluster and exported. For example, nearly half of all the carbon entering the olefins plant is exported from the industrial cluster as a CBB. This export of CBBs is also shown by the ethylene and propylene pipelines connecting the PoR cluster to other industrial clusters in the Netherlands, Belgium, and Germany.

#### CO_2_ Emissions

**Fig. 10:**
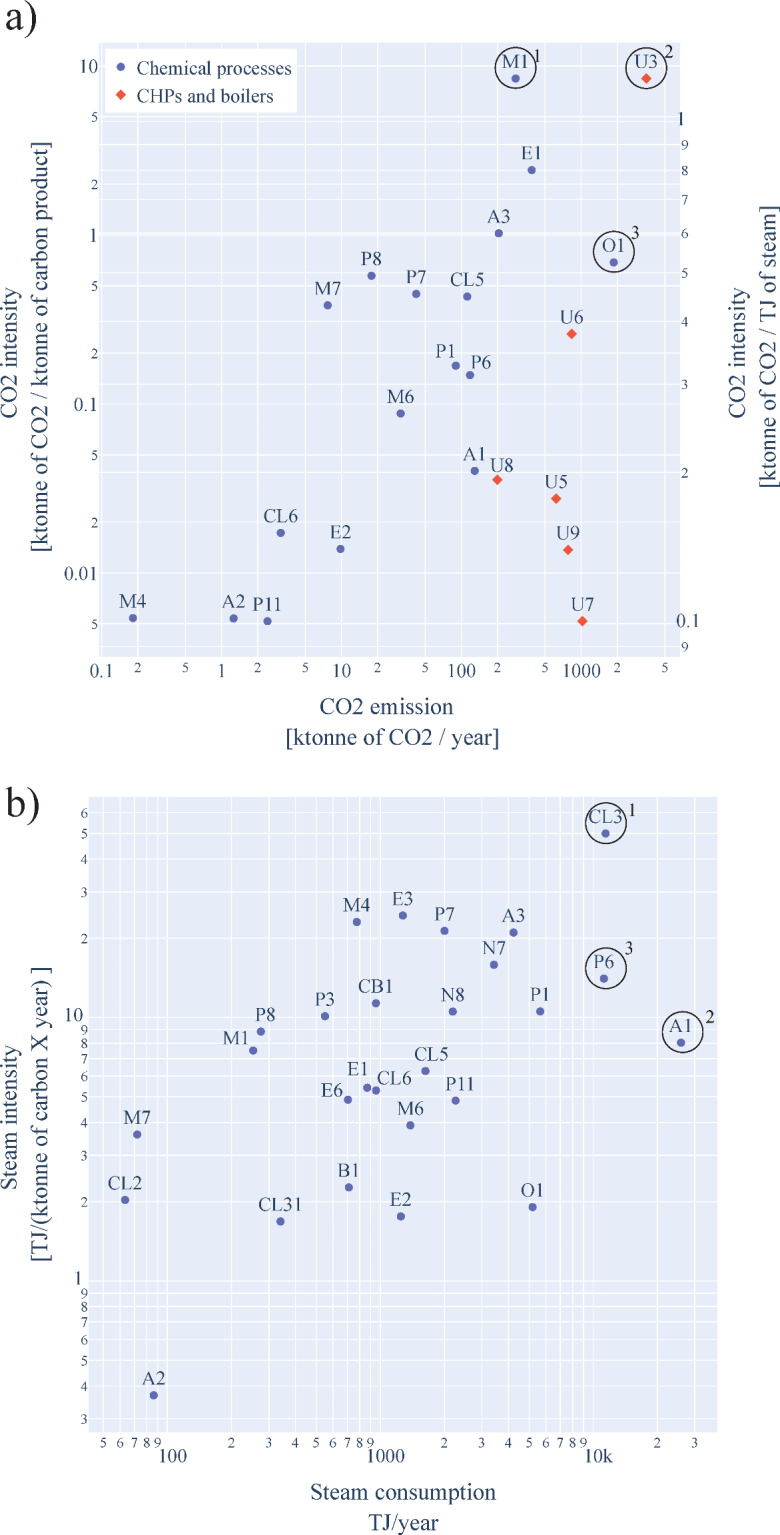
**a**) Logarithmic distribution of CO2 intensity over CO2 emission for the chemical processes (blue dots), boilers and CHPs of the RPC (red diamonds). **b**) Logarithmic distribution of steam consumption over steam intensity per process

In order to identify the processes with the highest CO_2_ contribution, a scatter plot was created, as depicted in Fig. 10a. It shows the CO_2_ intensity and emissions of the different processes in a scatter plot. The processes are depicted as blue dots, and the utility generation units are red diamonds. Overall, the RPC’s processes and utility generation units jointly emit 13Mt of CO_2_ per year. As shown in Fig. 10a, a significant part (64%) of the direct CO_2_ emissions originates from utility generation processes such as natural gas CHPs and the refinery gas CHP marked in Fig. 10a by 2 and 4, respectively. Note, however, that the CO_2_ emissions from the utility generation units have been summed up by their respective type of utility generation unit. In contrast, the CO_2_ emissions of the chemical processes are the direct emissions, including emissions produced solely by each process.

Additionally, from Fig. 10a, there appears to be a correlation between the CO_2_ emissions and the CO_2_ intensity. The olefins, marked by 3 in Fig. 10a, and the methanol plant, marked by 1, are the two most important deviations from this trend. For the olefins plant, this is mainly due to using part of its by-products as a fuel source to generate the required heat. As a result, it has the second highest amount of direct CO_2_ emissions overall and the highest direct CO_2_ emissions by a given chemical process. The methanol process uses natural gas as carbon feedstock and an energy source, resulting in high CO_2_ emissions. For most of the other processes, the CO_2_ emissions are mainly from burning lighter components.

When considering the CO_2_ emission intensity of the processes, the methanol process (M1) has the highest CO_2_ emission intensity. This results from the large-scale processes and, therefore, higher production capacities for CBBs. For instance, the olefins plant produces 3103 ktonne of products, while the methanol plant produces just 90 ktonne, resulting in a lower CO_2_ emission intensity for the olefins plant. When comparing the results of the CO_2_ emissions to other literature, similar results are obtained for the olefins plant. For instance, Flores-Granobles and Saeys [24] reported a similar CO2 intensity for a naphtha steam cracking based olefins plant.

Based on the results of the CO_2_ emissions, individual processes can be targeted to reduce their emissions, thereby having the most significant impact on the cluster’s overall CO_2_ emissions. For example, CO_2_ capture units could be installed for processes with high CO_2_ emissions, or the process technology could be changed to lower the CO_2_ emissions.

#### Steam Consumption and Steam Intensity

The RPC consumes about 95 PJ of steam per year and has a steam intensity of 15.2 TJ per ktonne of carbon product leaving the RPC. Figure 10b shows the cluster’s wide distribution of steam consumption and steam intensity. For instance, the PGME process (P3) has a steam intensity similar to the PO/TBA process (P1), while there is a significant difference in their respective steam consumptions. This difference in steam consumption can also be observed when comparing the phosgene process (CL2) and the olefins plant (O1). Therefore, there does not appear to be a correlation between the steam consumption of a process and its steam intensity.

The aromatics process (A1), marked by a 2, requires the highest amount of steam. This steam demand is due to its larger production capacity and the fact that most of the processing steps require an increase in temperature, thus obtaining all required heat from natural gas CHPs or boilers. Moreover, in contrast to the olefins plant (O1), it does not incinerate part of its byproducts to provide the heat required by the process. The other two processes that consume a large amount of steam are the MDI (CL3) and the PO/SM (P6) process, marked by a 1 and a 3 in Fig. 10b, respectively. For the PO/SM process, this is a result of the relatively large production capacity and a large number of distillation columns to purify the two products of the process. For the MDI process, this steam demand results from energy-intensive distillation steps to minimize chlorine-related losses and solvent recovery.

The steam intensity can be used to compare current processes with different production capacities. For instance, the MDI process (CL3) has the highest steam intensity. Notable is that MDI has a lower carbon content than other products in the cluster due to the presence of chlorine in the product. Additionally, the production capacity of the process (317 ktonne) is relatively low in comparison to the aromatics (1832 ktonne) and PO/SM process (962 ktonne). In combination with the relatively higher carbon content, this results in a high steam intensity for MDI and a lower steam intensity for the aromatics plant and PO/SM process.

Similar to the results of the CO2 emissions, the results of the steam consumption could be used to target individual processes. These targeted processes could reduce steam consumption by changing to more efficient alternative process technologies or improving heat integration.

## Conclusion

The introduction of alternative processes to replace the current fossil-based processes in a symbiotic petrochemical cluster can result in potential cascading impacts that can affect the material and energy exchange network and the overall performance of the cluster. As the currently available tools are insufficient to investigate this, there is the need first to understand the functioning of a petrochemical cluster, evaluate the potential impacts a petrochemical cluster might have, and assess its current performance. This paper uses a method that combines network analysis and technical, economic, and environmental assessment indicators to assess a petrochemical cluster’s performance. To illustrate the approach, a bottom-up representative model of a symbiotic petrochemical cluster based on the Port of Rotterdam industrial cluster was developed and analyzed.

This combined approach allows problematic processes to be identified and targeted for performance improvement by assessing key performance indicators. Meanwhile, the complex network properties allow the structure of the exchange network to be studied by assessing how interconnected the cluster’s processes are and how important their respective connections are. For instance, from the network properties, it can be concluded that only a few processes are strongly interdependent, with most of the processes having a lower interdependency. From the carbon analysis, it can be concluded that the aromatics and olefins processes are the most carbon-intense processes of the cluster. Therefore, the products of these processes are usually the focus of research when searching for more sustainable alternative options. However, as shown by the network analysis, replacing these processes is not straightforward as both processes are interdependent with other processes on the material layer. Replacing either of these processes could result in cascading impacts, potentially resulting in undesirable side effects.

These results show the importance of including existing interconnections between processes in petrochemical clusters when assessing the cluster’s performance. Additionally, the results illustrate the need for including these existing interconnections in assessing new or modified chemical processes. These interconnections are neglected during the performance assessment of new chemical processes using biomass, CO_2_, or plastic waste as feedstock. If these pre-existing connections are considered, the outcome of these assessments could significantly shift.

Based on this assessment method, the performance of a petrochemical cluster could be improved. First, a process could be targeted for potential improvement of its performance using the key performance indicators. The network analysis could then identify these modifications’ potential impacts on the overall cluster and assess their significance. This combined approach would allow for tailor-made solutions for transforming petrochemical clusters into more sustainable alternatives, where each transformed cluster has different processes and how they are interconnected.

This detailed approach to modeling a petrochemical cluster has, however, two caveats. First, it is resource-intensive. It requires highly detailed material and energy balances and, therefore, access to process-level data from companies or detailed chemical process models. It also requires data from exchanges between processes in an industrial cluster. These are often not readily available. Second, the embedded environmental footprint of the streams entering the cluster needs to be considered in the performance assessment and will have to be implemented to allow for a better understanding of the total environmental impact of the cluster. For instance, the performance could change if the footprint of carbon-intensive products entering the cluster is considered.

The presented approach and case study are focused on the material and energy interconnections between processes but could be further extended to monetary flows without changing the overall approach. Furthermore, additional environmental performance indicators could be used to extend the approach further. In future work, the model will be used to study the impacts of introducing alternative process options for producing chemical building blocks and end-of-value chain chemicals on the structure and performance of the cluster.

## Electronic Supplementary Material

Below is the link to the electronic supplementary material.

Supplementary Material 1

## Data Availability

The software used to model the petrochemical cluster is available at 10.4121/fa8a2ec4-7022-492c-852a-20a6bee61bcd and the input data is available at 10.4121/2103f9fb-2030-44a9-96f3-93a46ba2d545.

## Abbreviations

ASU: Air separation unit
CAPEX: Capital expenditure
CBB: Chemical building block
CHP: Combined heat and power
EB: Ethylbenzene
EDC: Ethylene dichloride
EVC: End-of-value chain chemical
GWP: Global warming potential
IC: Intermediate chemical
IS: Industrial Symbiosis
MDI: Methylene diphenyl diisocyanate
MTBE: Methyl tert-butyl ether
PFD: Process flow diagram
PGME: Propylene glycol methyl ether
PO: Propylene oxide
POR: Port of Rotterdam
PVC: Polyvinylchloride
RPC: Representative petrochemical cluster
SEC: Specific energy consumption
SM: Styrene monomer
TBA: Tert-butyl alcohol
VCM: Vinyl chloride monomer
