# Supplementary material for: Understanding the Level of Integration in Existing Chemical Clusters: Case Study in the Port of Rotterdam
Source: Circ Econ Sustain. 2024 Oct 9;5(1):125–46. doi: 10.1007/s43615-024-00410-5 (PMC11876245; doi:10.1007/s43615-024-00410-5)
Supplement: Supplementary file 1 — Supplementary Material 1 [file 43615_2024_410_MOESM1_ESM.docx]

**Supporting Information:**

**SI.1: Network properties**

To calculate the network properties, it is useful to represent the graph as a matrix. An adjacency matrix $A$ describes the network of a layer of the multiplex graph, where the entry $a_{ij}$ is equal to 1 when there is a link between nodes $i$ and $j$, and zero when there is no link. Similarly, a weighed adjacency matrix $W$ describes each layer while considering the weight of each link. In this weighted adjacency matrix, each entry $w_{ij}$ is equal to the weight of the link between nodes $i$ and $j$.

Based on the adjacency matrix, the importance of a node is determined by the number of connections a node has . The degree $k_{i}$ of a node $i$ is calculated following Equation (1):

$$k_{i}=\sum_{j\in N} a_{ij}$$

Where $N$ is the number of nodes. Additionally, the degree of centrality of a node can be normalized by the maximum number of possible links in the graph. This degree centrality of $C_{D,i}$ of a node $i$ can be determined by Equation (2):

$$C_{D,i}=\frac{\sum_{j\in N} a_{ij}}{N-1}$$

Alternatively, the importance of a node can be determined based on the weight of its links. Using the weighted adjacency matrix, this was done by calculating the weighted degree of a node or strength $s_{i}$ (Barrat et al., 2004) following Equation (3):

$$s_{i}=\sum_{j\in N} w_{ij}$$

These three network properties provide information on the importance of individual nodes on each layer of the multiplex graph in terms of the amount of interconnections and the weights of the connected links.

**SI.2: CAPEX calculations**

For each process, the bare equipment cost was determined with the Aspen Process Economic Analyzer. It uses the Aspen Plus process simulations to calculate the bare equipment costs. Based on these bare equipment costs, the total capital costs required for the construction of each process can be determined following chemical engineering design handbook of Towler & Sinnott (2013).

First, the cost of the plant itself or the inside battery limits (ISBL) costs are calculated by:

Where $C_{e}$ is the total bare equipment costs of a process, which were determined with the Aspen Process Economic Analyzer.

The cost for improving and modifying the infrastructure of the site (OSBL investment) is calculated by:

$$C_{OSBL}=C_{e}\cdot0.4$$

Based on the ISBL and OSBL costs, the engineering costs of the plant can be calculated by:

$$C_{EC}=\left(C_{ISBL}+C_{OSBL}\right)\cdot0.1$$

The variation from the cost estimate or contingency costs was calculated by:

$$C_{CON}=\left(C_{ISBL}+C_{OSBL}\right)\cdot0.1$$

The total capital costs or CAPEX is then determined by:

$$C_{CAPEX}=C_{ISBL}+C_{OSBL}+C_{EC}+C_{CON}$$

**SI.3 C: List of all the chemical and utility generation process**

Table A.1: List of all the chemical processes and their production capacities

| Process ID | Process name | Company | Product | Production [ktonne per year] |
| --- | --- | --- | --- | --- |
| A1. | Aromatics | L | Benzene | 892 |
|  |  |  | o-Xylene | 186 |
|  |  |  | p-Xylene | 754 |
| A2. | Cyclohexane | L | Cyclohexane | 273 |
| A3. | Purified terephthalic acid (PTA) | D | PTA | 34 |
| B1. | Biodiesel | E | Biodiesel | 388 |
|  |  |  | Glycerol | 46 |
| CB1. | Carbon black | P | Carbon black | 79 |
| CL1. | Chlorine | M | Chlorine | 820 |
|  |  |  | NaOH | 2367 |
|  |  |  | H_2_ | 28 |
| CL2. | Phosgene | N | Phosgene | 257 |
| CL3. | Methylene diphenyl diisocyanate (MDI) | N | MDI: 204 | 204 |
|  |  |  | Polymeric MDI (PMDI) | 113 |
| CL31. | Methylene dianiline (MDA) | N | MDA | 260 |
| CL4 | HCl | M | HCl | 412 |
| CL5. | Ethylene dichloride (EDC) / Vinyl chloride monomer (VCM) | O | VCMs | 612 |
|  |  |  | EDC | 80 |
| CL6. | Polyvinyl chloride (PVC) | R | PVC | 470 |
| CL8 | Chlorine recovery | M | HCl gas | 48 |
|  |  |  | HCl | 137 |
| E1. | Ethylene oxide (EO) | S | EO | 198 |
|  |  |  | EO water mixture: | 182 |
| E2. | Ethylbenzene (EB) | A | EB: | 784 |
| E3. | Ethylene glycol | S | Mono ethylene glycol (MEG ) | 113 |
|  |  |  | Di-ethylene glycol (DEG) | 16 |
|  |  |  | Tri-ethylene glycol (TEG) | 2 |
|  |  |  | Tetra-ethylene glycol (TEEG) | 1 |
| E6. | Polyethylene terephthalate (PET) | D | PET | 231 |
| M1. | Methanol | B | Methanol | 90 |
| M4. | Dimethyl ether (DME) | B | DME | 65 |
| M6 | Methyl tert butyl ether (MTBE) | I | MTBE | 400 |
|  |  |  | Isobutylene | 23 |
| M7 | Formaldehyde | K | Formaldehyde | 135 |
| N7. | Nitrobenzene | N | Nitrobenzene | 385 |
| N8. | Aniline | N | Aniline | 269 |
| O1 | Olefins | S | Ethylene | 878 |
|  |  |  | Propylene | 498 |
|  |  |  | Acetylene | 18 |
|  |  |  | Butene mixture | 93 |
|  |  |  | Butadiene | 116 |
|  |  |  | C9+ | 224 |
|  |  |  | Benzene | 689 |
|  |  |  | C7+ | 292 |
|  |  |  | Non aromatics | 295 |
| P1 | Propylene oxide (PO) / Tert butyl alcohol (TBA) | I | PO | 247 |
|  |  |  | TBA | 603 |
| P11. | Butane isomerization | I | Isobutane | 549 |
| P3. | PGME | I | PGME: 90 | 90 |
|  |  |  | Dipropylene glycol mono methyl ether (DPGME) | 11 |
| P6. | Propylene oxide (PO) / Styrene Monomer (SM) | A | PO | 296 |
|  |  |  | SM | 666 |
| P7. | Isopropyl alcohol (IPA) | Q | IPA | 105 |
|  |  |  | IPA Water | 60 |
| P8. | Acetone | Q | Acetone | 50 |
| U1. | Steam methane reformer | F | H_2_ | 100 |
| U2. | Air separation unit | H | Argon: 10 | 10 |
|  |  |  | O_2_ | 1395 |
|  |  |  | N_2_ | 4200 |

Table A.2: List of the utility generation processes and their production of steam and electricity

| Process ID | Process name | Company | LLPS [TJ/y] | LPS [TJ/y] | MPS [TJ/y] | HPS [TJ/y] | Electricity [TJ/y] |
| --- | --- | --- | --- | --- | --- | --- | --- |
| U3-C | Natural gas CHP | C | 0 | 671 | 601 | 522 | 1449933 |
| U3-DS | Natural gas CHP | D | 0 | 2013 | 0 | 0 | 1429393 |
| U3-FM | Natural gas CHP | N | 0 | 1417 | 2068 | 423 | 2537408 |
| U3-GS | Natural gas CHP | G | 2050 | 0 | 0 | 0 | 1429651 |
| U3-HS | Natural gas CHP | H | 2050 | 0 | 0 | 0 | 1429651 |
| U3-IS | Natural gas CHP | I | 2050 | 0 | 0 | 0 | 1429651 |
| U3-M | Natural gas CHP | M | 0 | 419 | 601 | 310 | 1446749 |
| U3-QS | Natural gas CHP | Q | 2050 | 0 | 0 | 0 | 1429651 |
| U3-SL | Natural gas CHP | S | 0 | 12161 | 0 | 0 | 2543652 |
| U5-Q | Petcoke CHP | Q | 0 | 2013 | 789 | 686 | 1265411 |
| U6-A | Natural gas boiler | S | 769 | 377 | 4248 | 0 | 0 |
| U6-E | Natural gas boiler | S | 705 | 0 | 507 | 0 | 0 |
| U6-I1 | Natural gas boiler | I | 854 | 0 | 0 | 0 | 0 |
| U6-I2 | Natural gas boiler | I | 854 | 0 | 0 | 0 | 0 |
| U6-M | Natural gas boiler | M | 0 | 1331 | 912 | 0 | 0 |
| U7-L | Refinery gas boiler | L | 0 | 0 | 10150 | 0 | 0 |
| U8-PL | Tailgas boiler | P | 0 | 0 | 1034 | 0 | 0 |
| U9-L | Refinery gas CHP | L | 0 | 5535 | 0 | 0 | 3166949 |

**References**

Barrat, A., Barthélemy, M., Pastor-Satorras, R., Vespignani, A., 2004. The architecture of complex weighted networks. Proc. Natl. Acad. Sci. U. S. A. 101, 3747–3752. https://doi.org/10.1073/pnas.0400087101

Towler, G., Sinnott, R., 2013. Chemical Engineering Design. Elsevier. https://doi.org/10.1016/C2009-0-61216-2
